# Hepatitis C (HCV) and Hepatitis Delta (HDV) Viruses in a Teaching Hospital in Southern Italy: What Is the Epidemiological Situation in the Era of New Drugs?

**DOI:** 10.3390/pathogens14090941

**Published:** 2025-09-17

**Authors:** Nadia Marascio, Grazia Pavia, Chiara Mazzei, Michele Manno, Giorgio Settimo Barreca, Cinzia Peronace, Carmela Ciurleo, Francesca Trimboli, Marta Pantanella, Angelo Giuseppe Lamberti, Giovanni Matera, Angela Quirino

**Affiliations:** Unit of Clinical Microbiology, Department of Health Sciences, “Magna Græcia” University of Catanzaro—“Renato Dulbecco” Teaching Hospital, 88100 Catanzaro, Italy; nmarascio@unicz.it (N.M.); chiara.mazzei@studenti.unicz.it (C.M.); michele.manno@unicz.it (M.M.); gbarreca@unicz.it (G.S.B.); cinzia.peronace@aourenatodulbecco.it (C.P.); carmela.ciurleo@studenti.unicz.it (C.C.); trimboli@unicz.it (F.T.); marta.pantanella@studenti.unicz.it (M.P.); alambert@unicz.it (A.G.L.); mmatera@unicz.it (G.M.); quirino@unicz.it (A.Q.)

**Keywords:** HCV, HDV, genotype/subtype distribution, screening tests, DAA, BLV

## Abstract

If the number of viral hepatitis infections is to be decreased worldwide, and the World Health Organization (WHO) elimination targets are to be achieved by 2030, this requires determining the burden of infection according to the WHO’s test-and-treat approach. In 2014, the introduction of Direct-Acting Antivirals (DAAs) revolutionized the management of Hepatitis C Virus (HCV); another improvement came in 2020, when the use of bulevirtide (BLV) was authorized as a treatment for chronic Hepatitis D Virus (HDV) infection, showing good efficacy. The present observational study was carried out between 2019 and 2024. The diagnosis of viral hepatitis was carried out by routine assays. HDV typing was performed by Sanger sequencing and phylogenetic analysis. Overall, the HCV antibody prevalence was 3.4% in the studied time span, and it was higher in males than in females (59% vs. 41%). In viremic patients, HCV1b (33%) and HCV2a/2c (25%) were the most common subtypes. The overall HCV viremic rate declined in 2022 (2.8%). Unlike HCV, 71.4% of HDV viremic patients were females, and they had a median age of 58 years. The viral load of HDV RNA ranged from 20 IU/mL to 8 million IU/mL. Viral genotypes were classified as HDV1c and HDV1e. In this study, we highlight the prevalence of HCV/HDV infections and their genotype evolution in Southern Italy, underscoring the urgent need to enhance screening and linkage to care. Finally, we quantify the burden of active infections in order to provide data from real-life settings, and we describe the virological status of people living with HCV or HBV/HDV, who may experience significant benefits in terms of liver-related mortality after DAA or BLV treatment.

## 1. Introduction

Each year, over 1.3 million deaths due to long-term complications of chronic viral hepatitis, such as cirrhosis and hepatocellular carcinoma (HCC), are still reported [1]. Hepatitis C Virus (HCV) infection and Hepatitis B Virus (HBV)/Hepatitis Delta Virus (HDV) coinfection or superinfection represent clinically significant challenges in the field of global viral hepatitis eradication, despite differences in pathogenesis and treatment strategies [2].

HCV still accounts for approximately 1.5 million new infections annually, with an estimated 58 million people living with the chronic disease, primarily due to healthcare procedures, injection drug use, and other parenteral exposures [3]. The introduction of safe and effective Direct-Acting Antivirals (DAAs) revolutionized HCV management, achieving a high sustained viral response (SVR) rate of 90–95% [4,5]; however, 20% of infections remain undiagnosed and untreated [1,3]. Furthermore, HCV exhibits substantial genetic heterogeneity, with at least eight genotypes and numerous subtypes influencing both epidemiology and treatment outcomes [6,7,8,9,10]. In Europe, HCV prevalence shows marked geographic variation, with rates ranging from 0.2 to 1.2% in Central and Northern European regions to 2.5–3.5% in Mediterranean countries [11]. Italy is recognized as having a higher HCV prevalence than the European average, especially in southern areas [12,13,14], with higher mortality rates from cirrhosis and HCC [15,16]. Despite ongoing surveillance efforts, reliable estimates of HCV infection prevalence are still unavailable. 

HDV coinfection and superinfection lead to faster progression to cirrhosis in nearly 80% of patients within 2–10 years [17], and carry an increased risk of HCC compared to HBV infection alone [18,19]. It is estimated that 12–72 million people are affected worldwide [18]. Among HBsAg-positive individuals in Europe, HDV seroprevalence is around 4.5% in general populations and up to ~16.4% in referral hepatology clinics, suggesting the presence of roughly 445,000 chronic HDV infections in Europe [18,20]. The introduction of HBV vaccination programs has played a pivotal role in reducing both the incidence and prevalence of HDV infection. In Italy, HDV antibody (Ab) positivity has varied over the decades, from 24.6% in the early 1980s down to ~8% in the late 1990s, and it has stabilized at around 6–10% in recent years [21]. In particular, HDV Ab positivity is approximately 6.4% among native Italians, whereas it can reach up to 26% in migrant populations, which underscores migration as an emerging challenge in the epidemiology of HDV infection [21,22,23]. HDV is classified into eight different genotypes and several subtypes, and the different clinical outcomes of infected patients may be related to genomic variability [24]. However, the actual prevalence of HDV infection is likely underestimated. The most recent guidelines from the European Association for the Study of the Liver (EASL) recommend HDV Ab testing in all HBsAg-positive patients [25]. Indeed, the implementation of HDV Ab reflex testing led to a fivefold increase in the absolute number of diagnosed HDV chronic cases [26,27]. Accurate prevalence data are essential for identifying patients that are eligible for novel antiviral therapies. In 2020, the use of bulevirtide (BLV) to treat chronic HDV infection was authorized by the European Medicines Agency (EMA), and it has shown good efficacy in patients with compensated liver disease [28].

In 2016, the World Health Organization (WHO) launched the Global Health Sector Strategy on Viral Hepatitis to reduce new infections and related deaths by 2030. Every year, on World Hepatitis Day (28th July), the WHO promotes new measures via public health programs to achieve the intended goal, improving screening tests, vaccination campaigns, and access to treatment worldwide [29]. In Italy, the number of HCV-treated patients has decreased since 2019, and without new extensive health strategies, by 2028, there will be even more undiagnosed infected individuals lacking care [30]. Monitoring treatment trends by country, by region, and globally, as previously assessed for HCV, is important, since the same model could be applied to HDV management, even if the HCV diagnosis rate was higher when new antivirals were launched [31]. Despite its clinical relevance, HDV infection was under-investigated for several years due to its limited therapeutic options. At present, the licensing of new antiviral drugs has revived interest in this satellite virus, encouraging researchers to conduct epidemiological studies to better define global prevalence and access to care [32]. The implementation of diagnosis schemes in the general population through national collaborations, as well as the sharing of prevalence data by hospitals, could help in evaluating the number of patient candidates for these new treatments, leading to a global reduction in clinical adverse outcomes and the potentially infectious reservoir [30,31,32]. 

In this scenario, the dynamic spread of HCV and HDV infections and their management in the era of new therapeutic options deserves renewed attention. Herein, we report a new consecutive diagnosis of HCV and HDV active infections according to antibody prevalence and genotype/subtype in order to provide an understanding of their evolution in the last six years. Finally, we identify viremic patients who may experience significant benefits in terms of liver-related mortality after treatment, contributing to the gathering of real-life data.

## 2. Materials and Methods

### 2.1. Ethical Statement

The study was conducted at R. Dulbecco Teaching Hospital, Catanzaro, Italy, and approved by the Ethics Committee of the Calabria Region (protocol code #311, 29 October 2024). Patient consent was waived due to the retrospective nature of the study and the inability to trace the patient’s identifier. Data was collected and treated in accordance with the Helsinki Declaration (64th WMA General Assembly, Fortaleza, Brazil, October 2013) and the principles of good clinical practice.

### 2.2. Antibody (Ab) and Viral Load Detection

Serum specimens were routinely collected between 1st January 2019 and 31st December 2024. Elecsys^®^ Anti-HCV II (Roche Diagnostics, Milan, Italy) was used to detect HCV Ab. HCV RNA viral load was determined by a cobas^®^ 4800 System HCV test (Roche Diagnostics, Milan, Italy), with a detection limit of 15 IU/mL. HDV IgM/IgG and HDV antigens were tested via enzyme-linked immunosorbent assay (ELISA, Dia.Pro. Diagnostic Bioprobes s.r.l, Sesto San Giovanni, MI, Italy). HBV antibodies and antigens were detected using commercial chemiluminescent assays (cobas^®^ 6000 Analyzer, Roche Diagnostics). HBV DNA viral load was quantified by real-time PCR, with a detection limit of 10 IU/mL (cobas^®^ 4800 System, Roche Diagnostics, Milan, Italy). HDV RNA viremia was revealed by RealStar^®^ HDV RT-PCR, with a detection limit of 9 IU/mL (Altona Diagnostics, Segrate, MI, Italy). 

### 2.3. HCV and HDV Genotyping

The 5′UTR and core regions of the HCV genome were analyzed by Versant HCV Genotype 2.0 Assay (Siemens, Healthcare Diagnostic Inc., Tarrytown, NY, USA). The HDV nucleic acid was extracted from 200 μL serum using the QIAmp viral RNA extraction kit (Qiagen, Hilden, Germany). Serum specimens of healthy subjects were used as negative controls. The cDNA was synthesized by the SuperScript™ VILO™ cDNA Synthesis Kit (Thermo Fisher Scientific Inc., Waltham, MA, USA). A partial region of the HDV small antigen (S-HDAg, 195 aa) was amplified using nested PCR by KAPA HiFi HotStart ReadyMix (Roche Diagnostics, Milan, Italy). PCR products were purified using the ExoSAP-IT™ Express PCR Product Cleanup (Applied Biosystems, South San Francisco, CA, USA). Sequencing reactions were performed through the Sanger method using the ABI PRISM 3500 genetic analyzer (Applied Biosystems, South San Francisco, CA, USA). Sequences were aligned with the MUltiple Sequence Comparison by Log-Expectation (MUSCLE) algorithm and manually edited via Molecular Evolutionary Genetics Analysis (MEGA) v.7 [33]. In order to classify genotypes/subtypes, the phylogenetic tree was constructed through the generalized time-reversible (GTR) nucleotide substitution model with γ-distribution using PhyML v3.0 (online version), including reference sequences according to Karimzadeh and colleagues [34,35]. Thousands of bootstrap replicates with the 70% cut-off were used to evaluate the reliability of phylogenetic clustering. The phylogenetic tree was visualized using FigTree v1.4.2 [36].

### 2.4. Statistical Analysis

The χ2 test was performed using GraphPad Prism version 4 for Windows (GraphPad Software, San Diego, CA, USA). The statistical significance level was set at a *p*-value < 0.05.

## 3. Results

### 3.1. HCV Antibody Prevalence over Time

**Figure 1:**
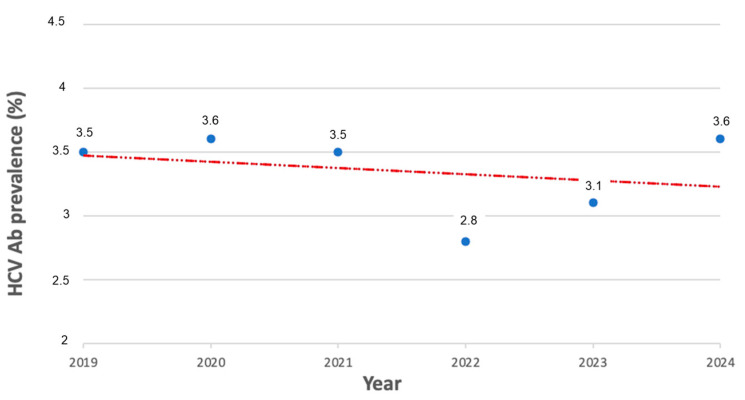
Trend in HCV Ab prevalence during the time span of the study. The number of samples screened each year was as follows: 2019 (n.11,560), 2020 (n.9476), 2021 (n.12,210), 2022 (n.11,852), 2023 (n.12,455), 2024 (n.9538). The trend over time is shown in blue dots.

From January 2019 to December 2024, 67,091 new consecutive subjects were screened for HCV infection. We found a heterogeneous distribution between males and females (59% and 41%, respectively). Overall, 3.4% of patients tested positive for HCV Ab and the prevalence remained stable throughout the 6 years (Figure 1).

### 3.2. HCV Genotype/Subtype Distribution

**Figure 2:**
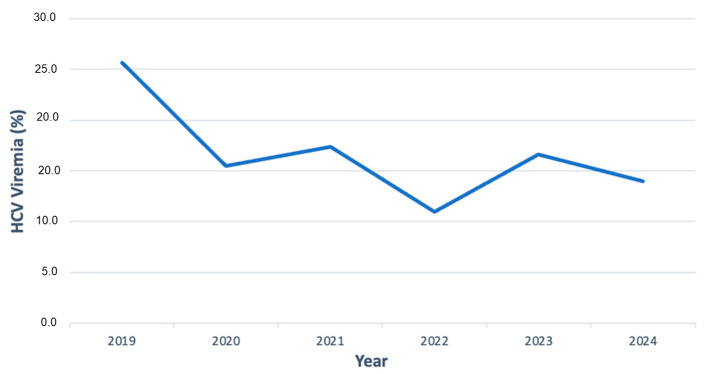
HCV viremia trend, including the cohort with active viral replication, from 2019 to 2024.

HCV genotype/subtype distribution was determined on 265 sera from as many viremic patients. The most prevalent genotype/subtypes were HCV1b (33%) and HCV2a/2c (25%) (Figure 2). The prevalence of viremic patients in the 6 years between 2019 and 2024 showed a decrease. Specifically, 26% (68/265) of viremic cases were identified in 2019, followed by 15% (41/265) in 2020, 17% (46/265) in 2021, 11% (29/265) in 2022, 17% (44/265) in 2023, and 14% (36/265) in 2024 (Figure 2).

**Figure 3:**
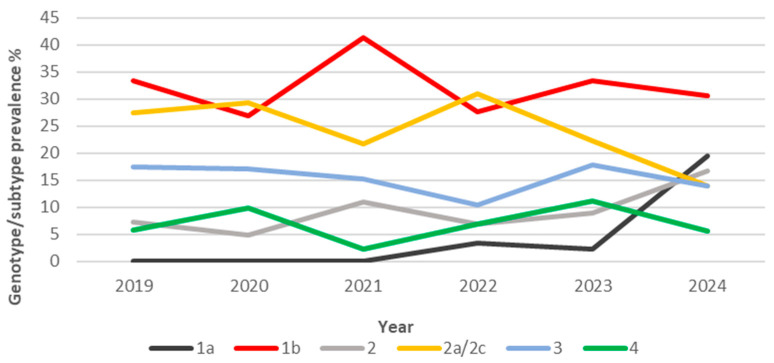
Dynamic prevalence of six most prevalent HCV genotypes/subtypes over time.

The decreasing trend was not statistically significant. Among the 265 serum samples, HCV RNA levels ranged from lower than the detection limit (<10 IU/mL) in one sample to 10^7^ IU/mL (19/265, 7%). The majority of chronically infected HCV patients had a viral load of 10^6^ IU/mL (120/265, 45%). The next most common viral loads were 10^5^ IU/mL (92/265, 35%) and 10^4^ IU/mL (24/265, 9%). No difference was recorded in median viremia according to HCV types. The most prevalent genotype/subtypes were HCV1b (33%) and HCV2a/2c (25%) (Figure 3). 

**Figure 4:**
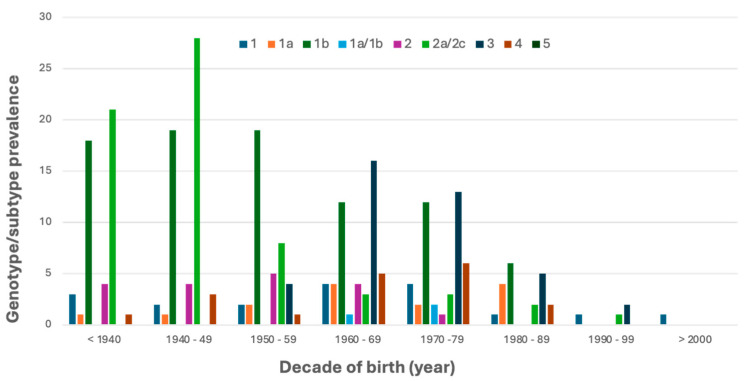
Distribution of HCV genotypes/subtypes according to the different decades of birth.

The pattern of HCV genotype/subtype prevalence showed a correlation with the decade of birth (Figure 4) and the gender (Table 1) of the general population, as males were more frequently infected (55.5%) than females (44.5%).

Most of the women who had detectable HCV RNA were born before 1949, while for men, the average age was lower, peaking in 1960–1969, demonstrating a substantial difference of about 20 years. Even though analyzing the data from patients born before 1949 results in the HCV2a/2c subtype e having the highest infection rate, statistical analysis showed no significant correlation between decade of birth and specific genotypes/subtypes. HCV2a/2c is more prevalent in females (66.2% vs. 33.8% males). In contrast, in later generations, there is a 23% increase in people infected with HCV3, and it is also more common in males (73.2%). Despite the small sample size, HCV1a and HCV4 genotypes were prevalent in males, at 100% and 72.2%, respectively. HCV1b and HCV2a/2c are the most represented regardless of patient age, with HCV1a showing a peak of 41.3% during 2021 and a non-significant change from 2019 (33.3%) to 2024 (30.6%); by contrast, the relative prevalence of HCV2a/2c decreased from 2019 (27.5%) to 2024 (13.9%), reaching its peak in 2022 (31%). HCV3 and HCV4 showed a median rate of 15.3% and 6.9%, respectively. 

### 3.3. HDV Antibody and Genotype/Subtype Prevalence

Between 2019 and 2024, a total of 65,971 hepatitis B surface antigen (HBsAg) tests were performed, in which 1804 individuals tested positive (2.7%). Overall, the annual HBsAg positivity rate was stable, ranging from 2.3% in 2021 to 3.1% in 2024. The reflex testing rates among HBsAg-positive individuals increased from 13.2% (2020) to 27.7% (2023). A total of 366 HDV antibody tests were performed over the six years; an increase in HDV positivity was noted from 2023 (2.9%, 3/103) to 2024 (10.1%, 7/69) (Supplementary Table S1). Real-time HDV-RNA RT-PCR was performed in 18/18 of HDV Ab-positive patients, and viremia was detected in 7/18 (38.8%) patients. The seven patients who had a confirmed HBV/HDV active coinfection, most of whom were female (71%, five out of seven), had a median age of 58 years (Table 2). 

The seven patients, two of whom were born in Italy, tested negative for HBeAg, while quantitative analysis of HBsAg showed marked variability, ranging from undetectable levels to 1400 IU/mL. HBV DNA viral loads were predominantly below the limit of detection (<10 IU/mL) in five patients, with only two subjects exhibiting low (405 IU/mL) to high (40,500 IU/mL) HBV viral load. HDV RNA level demonstrated a wide spectrum of viral replication activity, ranging from 20 IU/mL to over 8 million IU/mL. Genotypic analysis identified HDV1c (four of seven patients) and HDV1e (two out of seven patients). HDV was not genotypeable in one patient due to the low viral load (Table 2).

## 4. Discussion

### 4.1. Study Overview

This study represents a comprehensive evaluation of HCV/HDV antibody seroprevalence and genotype distribution in Southern Italy over the past six years. In addition, we estimated the proportion of chronic patients with active viremia who stand to benefit clinically from antiviral treatment, particularly by reducing liver-related morbidity and mortality. 

### 4.2. Update on HCV Prevalence and Genotype Distribution

From 2019 to 2024, a total of 67,091 consecutive individuals were screened for HCV infection, with an overall HCV antibody positivity rate of 3%. The prevalence remained relatively stable throughout the study period, though a gender imbalance was noted, as 59% of positive people were males and 41% were females. This finding shows an on-going decline when compared to our previous study covering the period 2008–2018 [37], which reported an overall higher seroprevalence of 4.9% among 120,009 tested individuals, with a decrease from 4.7% in 2008 to 3.6% in 2018. The gender imbalance is in agreement with our previous data, confirming a persistent male predominance in HCV infection rates across both decades [37]. This suggests that a substantial proportion of chronically infected patients remain either undiagnosed during acute infection or insufficiently linked to care, thus acting as a reservoir in the general population. The observations align with global data from the Polaris Observatory, which estimated that in 2022, only 20–30% of individuals with chronic HCV infection had initiated antiviral therapy [38]. Among the 265 patients with confirmed HCV viremia, most cases were diagnosed early into the timespan of the study; specifically, 26% of all cases were observed in 2019, followed by a significant drop to 15% in 2020 and 17% in 2021. The lowest percentage was reported in 2022 (11%), coinciding with the peak of the COVID-19 pandemic’s impact on healthcare services in the Calabria Region [39,40,41]. Although slight increases were observed in 2023 (17%) and 2024 (14%), these numbers have not returned to COVID-19 pre-pandemic levels [42]. In contrast, in the previously evaluated period (2008–2018), genotyping was performed on 1525 viremic patients, with no sharp interruptions observed in annual testing trends [37]. The local decline in diagnostic intensity and/or linkage to care during and after the pandemic was consistent with reports of decreased HCV testing and delayed treatment initiation across Europe [43]. This further underlines the long-term consequences of healthcare disruptions, especially in areas like Southern Italy, where older infected populations still account for a significant portion of the HCV burden. The most common genotypes/subtypes were HCV1b (33%) and HCV2a/2c (25%). These remain largely consistent with earlier data from 2008 to 2018, where HCV1b accounted for 47.2% and HCV2a/2c for 20.2% [37]. Notably, significant (*p*-value < 0.05) sex-related variations were observed: HCV2a/2c was more frequent in females (43/65) compared to males, whereas HCV3a showed higher prevalence in males (30/41), as well as HCV1a (9/9). This gender disparity aligns with prior reports linking genotype distribution to differential transmission routes and historical exposure risks [44,45]. HCV4 prevalence remained relatively stable across the two periods: 6.1% in the earlier study [37] versus a median of 6.9% in the current analysis. Additionally, the age stratification revealed a clear cohort effect. Women with detectable HCV RNA were predominantly born before 1949, and most of the infections were due to HCV2a/2c. Conversely, HCV-infected males tended to be younger, with infections peaking in those born between 1960 and 1969, showing a notable increase (23%) in HCV3 infections among later generations. This is consistent with data from 2008 to 2018 [37], in which genotype 1b was most common in those born before 1949, and HCV1a and HCV3 prevailed among individuals born after 1968. Genotype prevalence patterns also changed over time: HCV1a peaked in 2021 at 41.3%, showing only a slight, non-significant variation from 2019 (33.3%) to 2024 (30.6%). This reflects a marked increase compared to the previous period (2008–2018) [37], when HCV1a accounted for only 14.6% of genotyped cases. In contrast, HCV2a/2c decreased from 27.5% in 2019 to 13.9% in 2024, peaking in 2022 (31%). This differs from the 2008–2018 data [37], when HCV2a/2c represented 20.2% of genotyped infections and remained relatively stable throughout the decade. Genotypes HCV3 and HCV4 maintained a median prevalence of 15.3% and 6.9%, respectively. These findings likely reflect shifts in transmission modes over time, from blood transfusion and medical procedures in older cohorts to injecting drug use and other risk behaviors in younger populations, as previously documented [38]. HCV3 infection, particularly among patients with advanced liver disease, remains the most challenging to treat, due to slightly lower SVR rates reported compared to HCV1 and HCV2, as well as its being associated with rapid liver disease progression [46]. This is particularly relevant given the observed 23% increase in HCV3 infections among younger males in our real-life cohort population. The widespread availability of DAAs has revolutionized HCV treatment, with pan-genotypic regimens achieving SVR rates exceeding 90–95% [4,5]. Additionally, resistance-associated substitutions (RASs) account for approximately 5% of virological failures, emphasizing the ongoing importance of genotype testing and resistance profiling in selected cases [8,47]. Molecular analysis remains essential for identifying patients with RASs who may benefit from personalized treatment strategies [7,9,10,47]. 

### 4.3. First Survey on HDV Prevalence and Genotype Distribution

HBV/HDV coinfection or superinfection represents the most severe form of viral hepatitis, with a high risk of progression to cirrhosis and liver failure [48]. An early diagnosis of infection and routine HDV Ab screening significantly enhance case detection and improve clinical management [25,26]. The low testing coverage underscores the need for more systematic HDV screening among HBsAg-positive individuals. Owing to the EASL guidelines and to recently available therapies such as BLV, the therapeutic landscape for chronic HDV infection is changing [25]. In recent years, a substantial proportion of HBsAg-positive individuals have not been systematically screened [26,27]. From 2019 to 2024, a total of 65,971 HBsAg tests were performed, and 1804 individuals tested positive (2.7%). Despite annual fluctuations in the number of tests, HBsAg positivity rate remained generally stable (range 2.3–3.1%) over time, with the highest prevalence observed in 2024 (3.1%). Based on literature data, HBsAg carriers represented less than 2% until 2008, suggesting an increase in recent years because of shifting screening strategies to reduce the incidence of viral hepatitis. Interestingly, HDV prevalence does not always reflect a high prevalence of HBsAg [49]. In order to determine previous virus contact, reflex testing was carried out on serum samples taken from chronic HBsAg-carrying patients, and showed a small increase from 2021 (2.3%) to 2024 (3.1%). In our cohort, reflex testing showed a variation during the 2019–2024 time span. The low rate observed during the pandemic (13.2% in 2020) likely reflects delays in routine diagnostic services, while the subsequent increase to 27.7% in 2023 and 25.3% in 2024 may be linked to enhanced clinical awareness and the introduction of BLV in Italy [28]. Since March 2023, BLV monotherapy has been made available in Italy for people with detectable viremia and compensated cirrhosis. BLV is a subcutaneously administered entry inhibitor, blocking HDV/HBV access to hepatocytes via the sodium taurocholate co-transporting polypeptide (NTCP) receptor [50]. Overall HDV Ab positivity was 4.9%; in particular, the lowest prevalence was in 2021 (2.7%) and the highest was in 2024 (10.1%). This may reflect either an increase in the co-infection rates or an improved diagnostic process targeting at-risk individuals. In 2014, an Italian national study reported HDV Ab prevalence among patients admitted to several hospitals from 1987 (13.1%) to 2006 (8.7%), showing a progressive decline close to the trend of acute HBV infection; on the other hand, prevalence increased in 2014 (13.6%). Overall, antibody detection was higher in the Northern and Central regions (13.6%) than in the Southern and insular geographical areas (12.0%) [49,51]. In Italy, epidemiological surveillance showed a decrease in acute or chronic HDV cases in infected people aged 50 or more, and a vaccination campaign against HBV was introduced in 1991 [49,51]. In March 2025, the updated national surveillance program for acute viral hepatitis from the National Institute of Health (SEIEVA) reported that 1150 people tested negative for the major viral hepatitis, and only one case of HDV superinfection was found [52]. In our cohort, we found seven HBV/HDV co-infected patients with a median age of 58 years, mainly females (5/7, 71.4%). All of them were HBeAg-negative and HDV RNA-positive. Five out of seven patients were migrants, highlighting that the medical issues were related to immigrants coming from HDV-endemic areas [53]. In 2017, the HDV surveillance conducted by the Hepatitis Delta International Network (HDIN) in different regions worldwide (totaling 1576 patients) reported the infection to be more common in males (62%) and that the mean age was 36.7 years old. In particular, 77% of all screened patients were HBeAg-negative and 85.3% HDV RNA-positive [24]. HDV RNA viral loads spanned several logs, indicating heterogeneous viral replication profiles. HDV RNA quantification demonstrated a wide spectrum of viral replication activity, with levels ranging from 20 IU/mL to over 8 million IU/mL. HBsAg and HBV DNA quantification varied widely (Table 2). In a large cohort of 179 Australian patients, HBeAg presence was significantly inversely associated with HDV infection. The median HBV viral load was 180 IU/mL, less than for the HDV-negative subjects. HBV/HDV co-infection usually resulted in the suppression of virus B replication with lower levels of viraemia as the natural course of infection or due to HBV therapy [54,55]. A past study showed no evidence of correlation between HDV RNA levels and clinical markers (such as HBsAg) or stage of liver disease [56]. These data highlight the heterogeneity of viral replication, antigen expression in co-infected patients, and the importance of comprehensive virological monitoring in guiding clinical management and therapeutic decisions. Conventional PegInterferon alpha (PegIFNα) showed a suboptimal safety profile and therapy response rates (20–30%). Based on the identification of new virological targets, a novel antiviral treatment was developed to cure HDV infection [57]. Even if the efficacy of the new BLV therapy remained constant when treating the different HDV genotypes, they are responsible for different pathogenicities; HDV1 and HDV2 were associated with more severe disease, or with a more benign course of liver disease, respectively. As for HDV3, it was linked to poor patient prognosis. HDV classification should be performed to identify patients with a higher or lower risk of liver illness [24]. Among the circulating strains in the Calabria Region, we used phylogenetic analysis to identify two subtypes, HDV1c (4/7 patients) and HDV1e (2/7 patients). Very recently, Salpini and colleagues reported the epidemiological circulation of HDV genotypes/subtypes and their correlation with HDV liver disease severity. Nineteen patients with detectable viremia were infected by HDV1c (10/19) and HDV1e (9/19), highlighting the circulation of both subtypes in Central and Southern Italy. Interestingly, HDV1 circulation in Europe was correlated with a higher progression to cirrhosis than HDV strains circulation in other geographic areas [21]. 

### 4.4. Public Health and Clinical Implications

To better portray the virological complexity and clinical relevance of HCV, HBV, and HDV, dual or triple infections need to be considered due to shared routes of transmission and persistence within the same host [58,59,60,61]. As highlighted by Liaw et al. and further supported by Riaz et al. and Dwyre et al., such dual or triple infections are relatively common in high-risk populations and are often associated with more severe liver disease, including accelerated progression to cirrhosis and hepatocellular carcinoma [58,60,61]. Notably, co-infections do not result in a simple cumulative effect of pathogenicity; rather, they are defined by intricate inter-viral dynamics, including mechanisms of replicative interference and viral dominance, which can substantially influence the clinical course and treatment response [58,60,61]. In this regard, Jardi and colleagues reported that in dual HBV/HCV infections, HCV often exerts a stronger inhibitory effect on HBV replication, while in triple HBV/HCV/HDV infections, HDV becomes the dominant virus, more effectively suppressing HBV than HCV [59].

The observed decreasing trend of viral hepatitis incidence likely reflects the cumulative impact of public health interventions, including the discontinuation of reusable glass syringes, expanded screening efforts, and the changing landscape of infection in the era of new therapeutic options [28,29,62]. Based on our previous community-based survey and screening program on viral hepatitis in the Calabria region, this study is focused on HCV and HBV/HDV patients admitted to the Teaching Hospital, including high-risk groups, such as injection drug users, children born to hepatitis virus-positive mothers, contacts of infected patients, subjects with multiple sexual partners, and immigrants, as well as hospitalized subjects with other comorbidities or liver diseases [63,64]. Furthermore, people living with chronic HCV infection were mainly elderly and unaware (33.3%) of their infection [64]. In the present study, the percentage of the younger population (>2000 decade of birth) screened for HCV infection was 2.9% (1939/67,091). Enhancing screening and surveillance in targeted birth cohorts is essential for a public health strategy that aims to reduce transmission and promote long-term population health. HCV SVR following DAA therapy was associated with a lower risk of cirrhosis-related complications when cured promptly [65].

In the group of HBV/HDV coinfected or superinfected patients, HDV Ab and HDV RNA double reflex testing was ordered for 19.9% of HBsAg-positive patients and the total number of HDV Ab-positive patients, respectively. Five out of seven patients were born abroad, where vaccinating for HBV is not mandatory. In the era of globalization, an increase in diagnostic assays in low- and middle-income countries and among immigrant populations from endemic regions is necessary to fill in these critical data gaps so as to better assess the real global burden of HDV infection [66]. Despite the education campaigns, the number of voluntary tests remains suboptimal, emphasizing the need to implement systematic HCV screening and HDV double reflex testing to ensure timely diagnosis, linkage to care, and access to treatment. Since the discovery of HDV, viral infection has been significantly associated with a higher risk of cirrhosis and liver transplantation [54]. BLV could be an effective therapy for people with a new diagnosis or a history of PegIFNα treatment, following virological relapse.

The limitation of this study need to be addressed: this single-center observational study does not provide information on risk factors and linkage to care.

## 5. Conclusions

The stable HCV antibody prevalence of 3.4% over six years, alongside its viremic rate, highlights ongoing transmission and underdiagnosis. The observed shifts in genotype distribution, namely the predominance of HCV1b and HCV2a/2c with the increase in HCV3 infections in younger males, reflect an evolving epidemiology influenced by demographic and transmission changes. Our findings underscore persistent gaps in the HCV testing cascade, despite the availability of highly efficacious pan-genotypic DAAs that achieve SVR rates above 90%. Similarly, despite using BLV to treat chronic HDV infection showing good efficacy in patients with compensated liver disease, the application of reflex testing in all individuals who tested positive for HBsAg has not yet been implemented. Closely linked to diagnostic assays, the identification of HDV subtypes (such as 1c and 1e) may provide a better understanding of their pathogenesis, improve therapy responses, and better estimate their prevalence in specific geographical areas. To achieve the WHO’s target of viral hepatitis elimination by 2030, a running update of epidemiological surveillance is necessary in order to enhance screening programs and access to treatment. Finally, individualized therapeutic strategies based on the actively replicating virus and specialized care pathways are paramount to the achievement of optimal clinical outcomes.

## Figures and Tables

**Table 1:** HCV genotype/subtype distribution by gender considering the time span of 6 years.

Genotype/Subtype	Male		Female		*p*-Value
	N.	(%)	N.	(%)	
1	11	(61.2)	7	(38.8)	ns
1a	9	(100)	0	(0)	*p* < 0.05
1b	45	(51.7)	42	(48.3)	ns
1a/1b	3	(100)	0	(0)	ns
2	14	(58.3)	10	(41.7)	ns
2a/2c	22	(33.8)	43	(66.2)	*p* < 0.05
3	30	(73.2)	11	(26.8)	*p* < 0.05
4	13	(72.2)	5	(27.8)	ns
**Total**	**147**	**(55.5)**	**118**	**(44.5)**	ns

Legend: ns = not significant. Note: genotype 1 included not subtypeable (unknown subtype) HCV strains, and subtype 1a/1b included HCV strains not subtypeable as “a” or “b” by Versant HCV Genotype 2.0 Assay (Siemens, Healthcare Diagnostic Inc., Tarrytown, NY, USA).

**Table 2:** Demographic characteristics and virologic data of the seven HBV/HDV chronic coinfected patients included in the study.

**Demographic characteristics**							
**Patient ID**	**ID1**	**ID2**	**ID3**	**ID4**	**ID5**	**ID6**	**ID7**
Age	61	58	49	61	54	39	59
Gender	Female	Female	Male	Female	Male	Female	Female
							
**Virological characteristics**							
**HBcAb/HDAb**	R	R	R	R	R	R	R
**HBeAg**	NR	NR	NR	NR	NR	NR	NR
**qHBsAg (IU/mL)**	5.81	1.74	3.78	19.16	20	1461	-
**HBV DNA (IU/mL)**	<10	<10	<10	<10	450	40,500	<10
**HDV RNA (IU/mL)**	162,470	60,159	752,739	8,203,800	20	5114	245,960
**HDV Genotype**	1c	1e	1e	1c	NG	1c	1c

**Legend:** R—reactive, NR—non-reactive, NG—not genotypeable, q—quantitative.

## Data Availability

Newly generated were submitted to GenBank^®^ database. Sequences can be retrieved under accession numbers: PV936584-PV936589. Materials supporting the findings of this study are available within the article.

## Supplementary Materials

The following supporting information can be downloaded at: https://www.mdpi.com/article/10.3390/pathogens14090941/s1, Table S1: HBV/HDV markers of tested patients during the 2019–2024 time-span.
