# Supplementary material for: Hepatitis C (HCV) and Hepatitis Delta (HDV) Viruses in a Teaching Hospital in Southern Italy: What Is the Epidemiological Situation in the Era of New Drugs?
Source: Pathogens. 2025 Sep 17;14(9):941. doi: 10.3390/pathogens14090941 (PMC12472279; doi:10.3390/pathogens14090941)
Supplement: Supplementary file 1 [file pathogens-14-00941-s001.zip › pathogens-3760659-supplementary.pdf]

Supplementary Table S1. HBV/HDV markers of tested patients during the 2019–2024 time-span

**Years**<br><br>**Virological Markers**	**2019**	**2020**	**2021**	**2022**	**2023**	**2024**
HBsAg positive, n. (%)	295 (2.6)	265 (2.8)	278 (2.3)	321 (2.7)	372 (3)	273 (3.1)
HBsAg total test performed, n.	11220	9518	12247	11852	12392	8742
*rate of reflex-test, %	22.7	13.2	13.3	17.1	27.7	25.3
HDV Ab positive, n. (%)	3 (4.5)	2 (5.7)	1 (2.7)	2 (3.6)	3 (2.9)	7 (10.1)
HDV Ab total test performed, n.	67	35	37	55	103	69

Legend = *The rate of the reflex test was calculated considering the performed HDV Ab assay based on the HBsAg positive test.
